# IgA and FcαRI: Pathological Roles and Therapeutic Opportunities

**DOI:** 10.3389/fimmu.2019.00553

**Published:** 2019-03-22

**Authors:** Annelot Breedveld, Marjolein van Egmond

**Affiliations:** ^1^Department of Molecular Cell Biology and Immunology, Amsterdam UMC, Amsterdam, Netherlands; ^2^Amsterdam Infection and Immunity Institute, Amsterdam UMC, Amsterdam, Netherlands; ^3^Department of Surgery, Amsterdam UMC, Amsterdam, Netherlands

**Keywords:** IgA, CD89, mucosa, autoimmunity, IgA deficiency, microbiome, vaccination, therapy

## Abstract

Immunoglobulin A (IgA) is the most abundant antibody class present at mucosal surfaces. The production of IgA exceeds the production of all other antibodies combined, supporting its prominent role in host-pathogen defense. IgA closely interacts with the intestinal microbiota to enhance its diversity, and IgA has a passive protective role via immune exclusion. Additionally, inhibitory ITAMi signaling via the IgA Fc receptor (FcαRI; CD89) by monomeric IgA may play a role in maintaining homeostatic conditions. By contrast, IgA immune complexes (e.g., opsonized pathogens) potently activate immune cells via cross-linking FcαRI, thereby inducing pro-inflammatory responses resulting in elimination of pathogens. The importance of IgA in removal of pathogens is emphasized by the fact that several pathogens developed mechanisms to break down IgA or evade FcαRI-mediated activation of immune cells. Augmented or aberrant presence of IgA immune complexes can result in excessive neutrophil activation, potentially leading to severe tissue damage in multiple inflammatory, or autoimmune diseases. Influencing IgA or FcαRI-mediated functions therefore provides several therapeutic possibilities. On the one hand (passive) IgA vaccination strategies can be developed for protection against infections. Furthermore, IgA monoclonal antibodies that are directed against tumor antigens may be effective as cancer treatment. On the other hand, induction of ITAMi signaling via FcαRI may reduce allergy or inflammation, whereas blocking FcαRI with monoclonal antibodies, or peptides may resolve IgA-induced tissue damage. In this review both (patho)physiological roles as well as therapeutic possibilities of the IgA-FcαRI axis are addressed.

## Introduction

Immunoglobulins have important functions in immunity (1, 2). In mucosal areas like the gastrointestinal, genitourinary, and respiratory tracts, IgA is the predominant antibody present (3). It plays a key role at these surfaces, which are continuously exposed to antigens, food, and (commensal) microorganisms. Keeping a tight balance by tolerating commensals and harmless (food) antigens on the one hand and providing protection against harmful pathogens on the other hand is a challenging role of mucosal immunity. Mucosal IgA protects the host by diversifying the microbiota, neutralizing toxins and viruses, blocking colonization and penetration of pathogenic bacteria, clearing unwanted particles, and promoting sampling of antigens (4). Mucosal IgA is generally considered as a neutralizing, non-activating antibody. In serum, IgA is the second most abundant antibody after IgG. In contrast to mucosal IgA, the roles of serum IgA are relatively unexplored (5).

IgA deficiency is a relatively common disease with limited effects on human health (6), which supported the notion of IgA as a non-inflammatory antibody. By contrast, increased serum IgA levels or IgA autoantibodies have been reported in multiple (inflammatory) diseases including IgA nephropathy (IgAN), IgA vasculitis, dermatitis herpetiformis, celiac disease, inflammatory bowel disease (IBD), Sjögren's syndrome, ankylosing spondylitis, alcoholic liver cirrhosis, and Acquired immunodeficiency syndrome (7–9), although the role of IgA in pathology is still ill-understood.

IgA can interact with several receptors that are present on a variety of (immune) cells. The polymeric immunoglobulin receptor (pIgR) is present on the basolateral side of epithelial cells and mediates transport of dimeric IgA (dIgA) to the mucosal lumen, where it is released as secretory IgA (SIgA) (10). Retrograde transport of SIgA immune complexes back into the lamina propria can occur via the transferrin receptor (Tfr or CD71) on epithelial cells or through microfold cells (M cells), possible via interaction with Dectin-1 (11, 12). It has been reported that SIgA immune complexes in the lamina propria can be taken up by sub-epithelial dendritic cells (DCs) through interaction with Dendritic Cell-Specific Intercellular adhesion molecule-3-Grabbing Non-integrin (DC-SIGN) (13). B cells were shown to express the inhibitory IgA receptor Fc receptor-like 4 (FcRL4), which was suggested to contribute to the regulation of mucosal IgA responses (14). In the liver, hepatocytes express the asialoglycoprotein receptor (ASGPR) that is involved in clearance of IgA from the circulation (15). Mesangial cells in the glomeruli of the kidneys express CD71 and the recently identified β-1,4-galactosyltransferase as potential receptors to clear IgA (16). Furthermore, an Fc alpha/mu receptor is expressed in gut, spleen and lymph nodes, although its functions remain to be elucidated (17). Finally, the IgA Fc receptor FcαRI (CD89) is expressed by myeloid cells (18). It was demonstrated that interaction of monomeric serum IgA with FcαRI induces inhibitory signals (19, 20). As such, it is thought that IgA has an anti-inflammatory role during homeostatic conditions. By contrast, IgA immune complexes bind avidly to FcαRI, resulting in cross-linking and induction of pro-inflammatory responses, which may play an important role in resolving (mucosal) infections. Additionally, it was suggested that the presence of excessive IgA immune complexes can lead to uncontrolled and disproportionate immune cell activation, which leads to severe tissue damage in autoimmune diseases, such as IgA blistering diseases and rheumatoid arthritis (RA) (18). The number of diverse IgA receptors, their differential expression on cells and the distinct IgA-induced effector functions support the complex roles of this antibody in maintaining homeostatic conditions, which go beyond the dogma of IgA as a non-inflammatory regulator of mucosal immunity. This is further supported by the fact that several bacteria have evolved to escape IgA-mediated functions by for instance producing IgA1 proteases or expressing molecules that hamper interaction with IgA receptors (21–23).

This review addresses the different functions of IgA, its interaction with FcαRI,—as the best characterized IgA receptor -, in health and disease and the possibilities to target either molecule for therapeutic strategies.

## Structure of IgA

**Figure 1:**
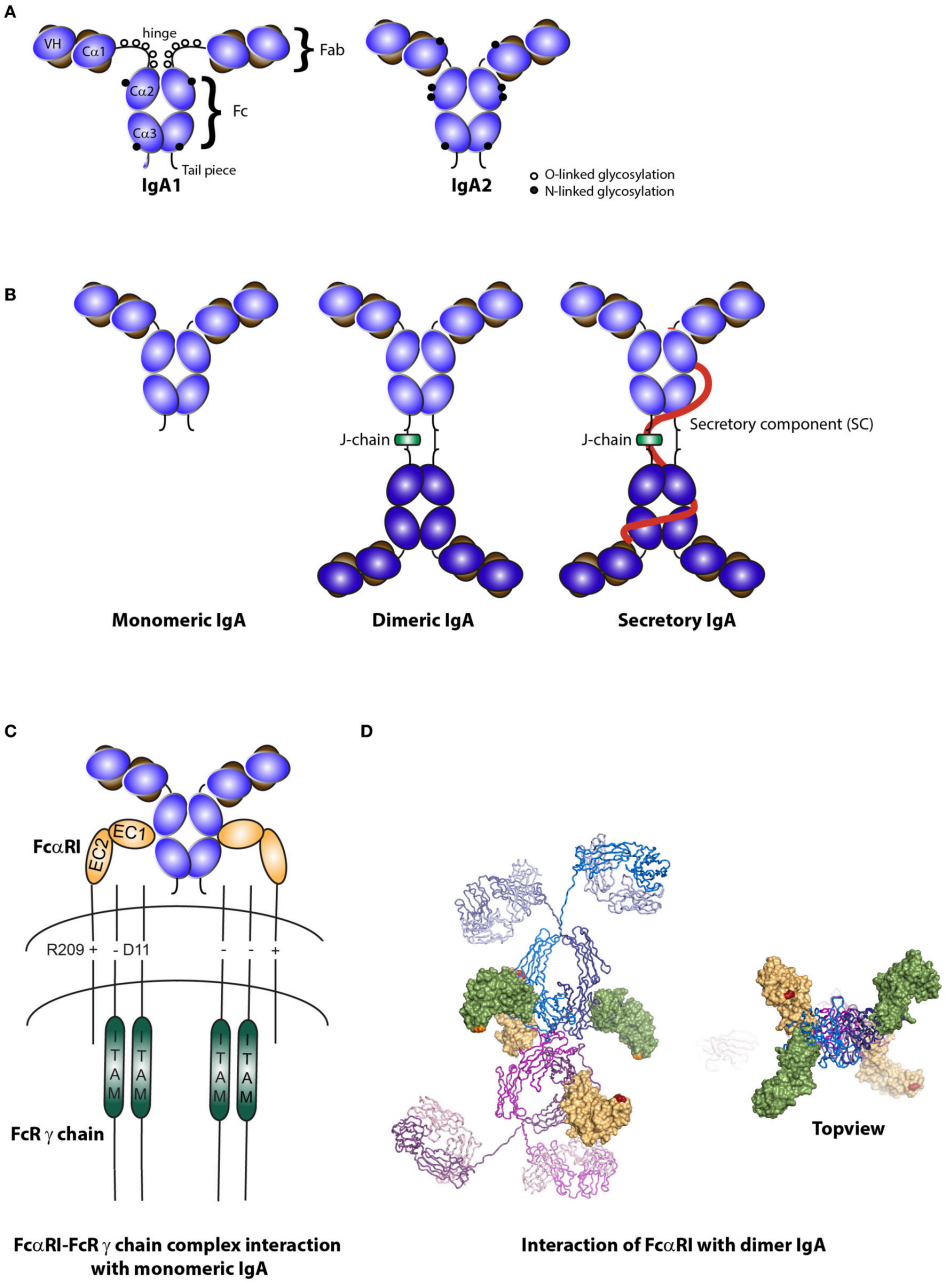
Structure of IgA isotypes and the IgA Fc receptor (FcαRI). **(A)** IgA1 vs. IgA2. IgA consists of two heavy chains (blue), each composed of three constant regions and one variable region, and two light chains (brown) that consist of one constant and one variable region. IgA1 contains a hinge region with O-linked glycosylation. **(B)** Monomeric, dimeric IgA, and secretory IgA. Dimeric IgA consist of two IgA molecules that are linked with a J-chain (green). Secretory IgA contains an additional molecule, the secretory component (SC; red). **(C)** Structure of FcαRI. FcαRI consist of a transmembrane domain, a short cytoplasmic tail and two extracellular domains (EC1 & EC2). It is associated with the signaling FcR γ chain via an electrostatic interaction. The IgA heavy chain junction of Cα2 and Cα3 binds to the EC1 domain of FcαRI in a 2:1 stoichiometry. **(D)** A model of dIgA1 bound to four FcαRI molecules. The FcαRI:IgA1-Fc complex (PDB 1OW0) was superimposed onto the solution structure of dIgA1 published by Bonner et al. (24) (PDB 2QTJ). FcαRI molecules bound to the top or bottom IgA1 antibodies are colored green or yellow, respectively. The C-terminal residue of each receptor is shown in orange or red to illustrate the membrane-proximal region. Kindly provided by Andrew B. Herr, PhD (Cincinnati Children's Hospital).

IgA is the most predominant antibody produced by humans with a synthesis rate of 66 mg/kg each day. IgA can be subdivided into IgA1 and IgA2 (Figure 1A). The IgA1 hinge region contains multiple O-linked glycans and two N-linked glycosylation sites per heavy chain. The truncated hinge of IgA2 lacks O-glycans and each heavy chain contains two additional N-glycans (25).

IgA1 comprises 80–85% of total human serum IgA (1–3 mg/ml) and is prevalent on many mucosal surfaces including the nasal, bronchial, gastric, and small intestinal mucosa. IgA2 is predominantly present in the colon (26). This division of IgA1 and IgA2 between the small and large intestine may reflect the luminal distribution of food proteins and gram-negative bacteria, which is supported by findings that bacterial overgrowth in the small intestine shifts production toward IgA2 (27). Whereas, IgA1 is susceptible to proteolytic cleavage due to a longer hinge region, IgA2 is resistant to bacterial proteases that are present in the lumen of mucosal areas (6, 21).

Serum IgA is predominantly monomeric in humans and primates (while dimeric in other animals) and is produced by local plasma cells in the bone marrow, spleen, and lymph nodes. IgA at mucosal surfaces is produced by local plasma cells as dimeric molecules, although small amounts of monomers, trimers and tetramers or polymers can also be present (1, 3). Dimeric IgA is composed of two monomers, which are linked with a J-chain (Figure 1B). It associates with the pIgR present at the basolateral side of epithelial cells in mucosal surfaces, after which dIgA is transported across the epithelium and released into the lumen. For every molecule of dIgA that is transported across the epithelium, one pIgR is needed (10, 28, 29). At the luminal side, pIgR is cleaved and a part, referred to as secretory component (SC), remains attached thereby forming SIgA (Figure 1B). Secretory component is a hydrophilic and highly (N- and O-linked) glycosylated negatively charged molecule, which protects SIgA from degradation when present in luminal secretions (30). Glycan removal led to reduced binding capacity of SIgA to gram-positive bacteria, indicating the essential role of carbohydrate structures in efficient IgA coating of (mainly commensal) bacteria (31).

## Structure and Expression of FcαRI

FcαRI is a member of the Fc receptor immunoglobulin superfamily (32). It is expressed on cells of the myeloid lineage, including neutrophils, eosinophils, monocytes, macrophages, Kupffer cells, and certain DC subsets (18, 33, 34). Additionally, FcαRI expression on human platelets was described (35). Unlike for other Fc receptors, the FcαRI gene is located on chromosome 19 (19q13.4) within the leukocyte receptor cluster (LRC). This region also encodes natural killer cell inhibitory/activatory receptors and leukocyte Ig-like receptors. The FcαRI amino acid sequence has therefore more similarity with these receptors than with other Fc receptors (19, 36). The EC1 and EC2 exons each encode an extracellular Ig-like domain. These domains are folded in an angle of ~90°. The TM/C exon encodes both the transmembrane domain and a short cytoplasmic tail (Figure 1C) (36).

Surface expression of two isoforms of FcαRI has been described on human phagocytes. The a.1 isoform is a 32 kDa single pass transmembrane receptor, which can be extensively glycosylated (six N-linked sites and seven O-linked sites) (37). The molecular weight of the a.1 isoform varies between 55 and 75 kDa on neutrophils and monocytes, while the molecule is heavier (70–100 kDa) on eosinophils due to more extensive glycosylation (21, 37). The a.2 isoform misses 66 nucleotides in the EC2 exon resulting in loss of one O-linked glycosylation site and has a weight of 28 kDa (without glycosylation) (38). The a.2 isoform is solely expressed on alveolar macrophages (38).

The expression of FcαRI was estimated to represent 66,000 and 57,000 molecules on neutrophils and monocytes, respectively (33). Expression level can be modulated by several mediators. On neutrophils, N-formylmethionyl-leucyl-phenylalanine (fMLP), interleukin (IL)-8, tumor necrosis factor-alpha (TNF-α), lipopolysaccharide (LPS), and granulocyte-macrophage colony–stimulating factor (GM-CSF) enhanced the expression of FcαRI (39–41). GM-CSF has also an important role in mobilizing neutrophils from the bone marrow (42) and was shown to induce high affinity binding of IgA by human neutrophils (43). Neutrophil FcαRI upregulation was rapid and either induced by *de novo* synthesis or via transport from an intracellular pool to the cell surface (44). On monocytes and monocyte-like cell lines FcαRI expression was enhanced by calcitriol, LPS, TNF-α, GM-CSF, and IL-1β, while downregulation was observed in response to transforming growth factor-beta (TGF-β) or interferon-gamma (IFN-γ) (45, 46). Both monomeric and, to a greater extent, polymeric IgA were able to downregulate FcαRI, possibly due to receptor aggregation, resulting in internalization (47–49).

## IgA and FcαRI

### Binding of IgA to FcαRI

FcαRI is a low affinity Fc receptor for monomeric IgA and dIgA (K_a_ = 10^6^ M^−1^), while IgA immune complexes bind with high avidity and cross-link FcαRI (50). Monomeric IgA binds to the EC1 domain of FcαRI via its Cα2 and Cα3 domains in a 2:1 stoichiometry (i.e., one IgA molecule binds two FcαRI molecules) (Figure 1C) (51, 52). Presence of residues Pro440-Phe443 and Leu257-Leu258 in these domains is essential for IgA binding to FcαRI (53).

Dimeric IgA contains four FcαRI binding sites and can therefore theoretically bind four FcαRI, although this is presumably not possible due to steric hindrance (Figure 1D) (24). It remains to be elucidated how dIgA exactly interacts with the FcαRI. Binding of SIgA to FcαRI is hampered because of steric hindrance by SC. In order for SIgA to activate cells, co-stimulation of FcαRI, and the lectin Mac-1 (CD11b/CD18) was necessary (54).

Little is known about the difference between IgA1 and IgA2 binding to FcαRI (if any) or the influence of glycosylation on binding capacity. It was however shown that a specific mutation (Asn58 to Glu58) resulted in an altered glycosylation pattern of FcαRI, which increased the binding capacity of IgA nearly 2-fold (55). Removal of sialic acids led to a nearly 4-fold increase of IgA binding. This demonstrates the importance of glycosylation at position 58 of FcαRI in binding affinity for IgA (55). N-glycans located at the external surface of the IgA heavy chain were important for interaction with FcαRI as well (56). Furthermore, it was demonstrated that alterations in IgA1 glycosylation and impaired sialylation of FcαRI were linked to increased binding of IgA1 to FcαRI on neutrophils of patients with IgA nephropathy, which may influence pro-inflammatory functions (47). In transfectants, eosinophils, and monocytes FcαRI binding capacity for IgA immune complexes was enhanced by incubation with several cytokines like GM-CSF, IL-4, and IL-5, without affecting the expression level of the FcαRI on the cell surface (43, 57).

Competitive binding for FcαRI has been described for pentraxins, including the acute phase C reactive protein and serum amyloid P component, resulting in cell activation (58). These proteins are characterized by a pentameric ring-like structure containing five subunits, which recognize a similar site on FcαRI as IgA. However, mutations in FcαRI outside the IgA binding site did not affect IgA binding, but enhanced pentraxin binding 2-fold, suggesting that pentraxins bind to a broader region on FcαRI than IgA (58).

Importantly, *Staphylococcus aureus* and group A and B streptococci developed evasion strategies for IgA-mediated elimination by FcαRI-expressing immune cells by producing several decoy proteins that obstruct binding of IgA to FcαRI (22, 23). Anti-IgA proteins from group B streptococci include Sir22, Arp4 and an unrelated β protein, whereas *Staphylococcus aureus* produces staphylococcal superantigen-like protein seven. These proteins bind to specific Fc residues (amino acids Pro440-Phe443; i.e. PLAF) in the Cα2 and Cα3 domains of IgA (22, 23, 59). The fact that pathogens evolved and developed mechanisms to evade IgA-mediated elimination by FcαRI-expressing immune cells (60), challenges the dogma of IgA as a non-inflammatory and possibly redundant antibody.

### Cell Activation After FcαRI Cross-Linking

Cross-linking of FcαRI by IgA immune complexes (or IgA-opsonized pathogens) induces a variety of processes, including phagocytosis, antibody-dependent cellular cytotoxicity, superoxide generation, release of inflammatory mediators, and cytokines as well as antigen presentation (61–65). Due to hampered binding of SIgA to FcαRI, SIgA does not induce efficient uptake of pathogens by neutrophils or Kupffer cells. However, respiratory burst in neutrophils can be triggered by SIgA, although less efficiently compared to serum IgA (54). Phagocytosis of IgA-coated bacteria or yeast particles by neutrophils was enhanced after priming with GM-CSF or IL-8 (43, 66). Uptake of IgA-coated particles induces increased reactive oxygen species production, subsequently resulting in the release of neutrophil extracellular traps (NETs), which are web-like structures consisting of DNA and proteins (e.g., elastase and myeloperoxidase) able to catch pathogens (61). FcαRI cross-linking furthermore induces release of the neutrophil chemoattractant leukotriene B4 (LTB4) by neutrophils, thereby generating a positive migration feedback loop (64). LTB4 was also shown to mediate migration of monocyte derived DCs (67). As such, in case of mucosal infection neutrophils can function as potent phagocytes, which eliminate invading IgA-coated pathogens, and attract DCs that present antigens to T cells. It was recently shown that *in vitro* generated human mucosal CD103^+^ DCs (cultured with retinoic acid) express FcαRI. After stimulation with IgA-coated *Staphylococcus aureus* in the presence of Pam3CSK4 CD103^+^ DCs produced pro-inflammatory cytokines including TNF-α, IL-1β, IL-6, and IL-23, which was dependent on FcαRI (68). Furthermore, IgA-stimulated CD103^+^ DCs enhanced IL-17 production by allogeneic T cells and IL-22 production by intestinal type three innate lymphoid cells, respectively. This suggests that FcαRI crosslinking by IgA induces a pro-inflammatory T helper 17 response accompanied with IL-22 induced activation of epithelial cells, which might contribute to tissue repair (68).

### Signaling via FcαRI

In order to initiate optimal effector functions after FcαRI cross-linking, FcαRI needs association with the FcR γ-chain, which contains an immunoreceptor tyrosine-based activation motif (ITAM) in its intracellular domain (69–72). After cross-linking of FcαRI, the src family kinase Fyn aggregates with the receptor complex and phosphorylates the tyrosines in the ITAM in order to act as docking sites for signaling molecules (69, 73, 74). Recruited Syk associates with the phosphorylated ITAM and plays an essential role in the signaling cascade. It directly activates phosphoinositide 3-kinase and phospholipase Cγ, which are involved in two separate signaling routes. These pathways are interconnected and can therefore modulate each other (69, 72, 75). Eventually the signaling pathways will activate the cell, resulting in pro-inflammatory functions (18, 66). Alternatively, it was demonstrated that inhibitory signals can also be transduced via the FcαRI-FcR γ-chain complex. Monomeric targeting of FcαRI (not leading to cross-linking) results in an inhibitory signal and is dependent on Lyn instead of Fyn kinase (74). Phosphorylation of Syk, LAT, and ERK is hampered by “inhibisomes” that contain signaling molecules and both inhibitory and activating receptors (20, 76). Inhibisomes impair activating Fc receptor functioning via a process referred to as inhibitory ITAM (ITAMi) receptor signaling that initiates anti-inflammatory responses to dampen pro-inflammatory responses induced by other Fc receptors (77). Thus, Lyn and Fyn are essential molecules controlling ITAM inhibition and activation, respectively (74). Signaling via FcαRI was recently described in more detail in Aleyd et al. (18).

## IgA and FcαRI mediated functions at different locations

### In the Lumen of Mucosal Sites

**Figure 2:**
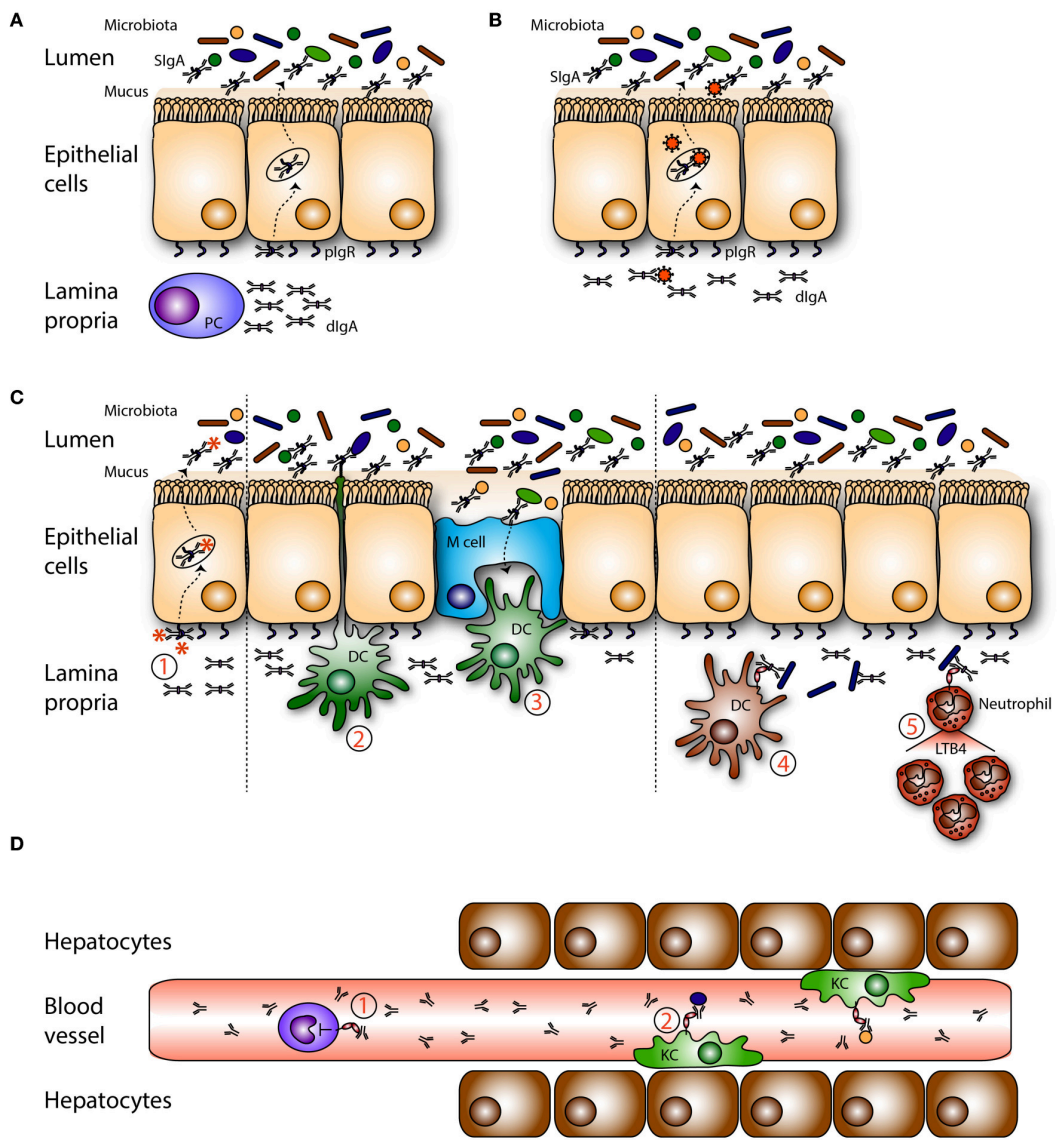
Roles of mucosal IgA in homeostasis. **(A)** Dimeric IgA (dIgA) is produced by local plasma cells (PC) in the lamina propria. Dimeric IgA is transported to the intestinal lumen by binding to the polymeric immunoglobulin receptor (pIgR) present on epithelial cells. In the lumen it is released as secretory IgA (SIgA) where it can coat (commensal) bacteria. **(B)** On route dIgA can bind, neutralize and eliminate viruses. **(C)** (1) Infiltrated antigens and pathogens are opsonized by dIgA and transported back into the lumen. Sub epithelial dendritic cells (DCs) can (2) sample antigens or (3) take up SIgA-coated pathogens that enter via microfold cells (M cells). Pathogens in the lamina propria are coated with dIgA after which this immune complex is taken up by FcαRI- expressing (4) DCs, and (5) neutrophils. In response neutrophils secrete leukotriene B4 (LTB4), hereby attracting more neutrophils, which will clear the infection. **(D)** Serum IgA is (1) capable of inhibiting (unwanted) pro inflammatory responses in the circulation via ITAMi signaling in monocytes. (2) IgA-opsonized bacteria which have leaked into the circulation are taken up by FcαRI-expressing Kupffer cells (KC) in the liver.

The functions of SIgA in the intestinal lumen have been well-characterized (Figure 2A). SIgA and commensal microbes have important roles in maintaining balance between tolerance toward non-harmful commensals and food compounds vs. immunity against pathogens (78, 79). When SIgA is released from the epithelial layer into the lumen, it remains attached to the outer mucus layer (80). This close localization to bacteria enables SIgA to disturb bacterial motility and to surround pathogens that have a hydrophilic shell. SIgA coating blocks entrance of pathogens into the intestinal epithelium (a process called immune exclusion). This coating leads to agglutination, because antibody-mediated crosslinking of surface antigens will lead to formation of bacterial clumps. Peristaltic bowel movements ensure removal of bacterial aggregates. Bacterial products like enzymes and toxins can be neutralized by SIgA and adherence to host cells, including epithelial cells, is prevented (78). Thus, together with the mucus layer, SIgA forms a barrier against pathogens, and commensals by preventing colonization and penetration of the mucosal epithelium, thereby avoiding infection and antigen leakage into the systemic circulation (81).

Recently the interplay between luminal microbiota and SIgA was investigated in more detail (4, 82) (Figure 2A). It is still unclear how SIgA and the microbiota exactly relate to each other, but it was described that SIgA shapes and diversifies the gut microflora on the one hand, while the microbiota on the other hand has an important role in regulating IgA levels (82–84). During weaning up to 70% of intestinal commensals are coated with SIgA in mice and the majority of human fecal bacteria in healthy donors are opsonized with IgA, emphasizing the importance of this association in maintaining homeostasis (31, 85). A wide variety of commensals can be opsonized with polyreactive IgA, although it remains unclear how these IgA antibodies are generated, what the relevant targets are and how binding affects the fitness and physiology of commensals (86). In contrast to commensals, pathogens elicit highly specific T-cell dependent IgA responses (86, 87). Genetic background was shown to influence the repertoire diversity and abundance of innate IgA as germ free (GF) naïve BALB/c mice already had substantial innate IgA levels, independent of microbiota colonization, while this was not the case for C57BL/6 mice (83).

Furthermore, highly glycosylated IgA was able to bind to several gram negative bacteria, which modulated their gene expression, leading to enhanced processing of fibers and production of butyrate as well as a diversified microbiota composition (82). Moreover, it was shown that the disbalanced microbiota of colitogenic mice became more balanced and diversified after oral administration of the monoclonal IgA W27, which is a high-affinity polyreactive antibody (84). Importantly, mice lacking IgA, J-chain or pIgR were unable to efficiently protect themselves against mucosal infections. These mice have an altered gut microbiota as well as increased mucosal permeability, which renders them more susceptible to develop colitis (80, 88–94). Of note, lack of J-chain or pIgR also led to absence of SIgM which may have contributed to altered microbiota and compromised immunity (88, 91, 94). A diversified microbiota can regulate the amount of SIgA present in luminal secretions by enhancing the level of pIgR on epithelial cells (1, 31, 95), illustrating the complex interrelationship between IgA (and IgM) and the microbiota in maintaining homeostatic conditions.

### In Epithelial Layers

It was described that SIgA can be translocated back into the lamina propria (retrograde transport). SIgA and SIgA immune complexes can be transported across specialized epithelial cells, referred to as M cells, which are located in the Peyer's patches of the intestine. Dectin-1 that is expressed by M cells may facilitate transepithelial transport (11, 96). Additionally, a role for TfR1 on epithelial cells in retrotransport of SIgA-coated gliadin was reported in studies investigating celiac disease (12). Retrotransported SIgA immune complexes can be taken up by DCs that are present in the subepithelial dome. *In vitro* models support an essential role for DC-SIGN in recognizing SIgA immune complexes (13). The retrograde transport mechanism and recognition of SIgA by DCs is thought to be essential in monitoring the antigenic status of the intestinal lumen.

On route through epithelial cells, dIgA has the ability to intercept and disarm viruses and redirect them into the lumen, after which they are removed with the feces (Figure 2B). Neutralization of several viruses by IgA including sendai, influenza, rota, measles, and human immunodeficiency virus (HIV), as well as bacterial LPS was demonstrated *in vitro* (97–104). IgA has enhanced neutralizing capacity compared to other Ig isotypes due to the N-linked glycosylation of position N459D in the heavy chain Cα3 (105). This effect is independent of the antigen binding site as glycosylated non-specific IgA neutralized the virus as well.

IgA deficient mice, J-chain deficient mice (lacking dIgA, SIgA, and SIgM) and pIgR deficient mice (lacking SIgA and SIgM) have defects in clearing viruses, are more susceptible to reinfection and lack protective immunity (106–108), which emphasizes the important role of IgA in anti-viral immune responses. By contrast, in a recent study pIgR knock-out mice showed a reduced acute norovirus infection rate, suggesting that pIgR and natural polymeric immunoglobulins may promote viral infections. It was suggested that the less diversified intestinal microbiota present in pIgR deficient mice was responsible for reduced infection. pIgR deficient mice had enhanced levels of the anti-viral cytokine IFN-γ, which might explain the reduced infection rates (109).

### In the Lamina Propria

Food particles and commensal microbiota are abundantly present in the lumen and can continuously reach the lamina propria through diffusion (via epithelial tight junctions) or transcytosis (Figure 2C). These non-harmful components need to be tolerated and they need to be cleared from the mucosa to avoid the formation of immune complexes, which may trigger undesired immune responses. In the lamina propria dIgA can eliminate both non-harmful and harmful components from the tissue by transporting immune complexes back into the lumen through association with the pIgR (97, 101). Additionally, FcαRI^+^ residing immune cells may help to clear IgA-opsonized pathogens. Under homeostatic conditions, only few FcαRI^+^ cells are found in mucosal areas (own unpublished data). However, as dIgA-opsonized complexes have the ability to cross-link FcαRI, leading to release of LTB4, neutrophils can be rapidly recruited (64) (Figure 2C). Furthermore, *in vitro* generated FcαRI^+^ CD103^+^ DCs were shown to produce pro-inflammatory cytokines after stimulation with IgA-coated *Staphylococcus aureus* in the presence of Pam3CSK4, suggesting that a protective adaptive immune response is initiated during mucosal infections (68).

### Systemic Protection

It was demonstrated that a variety of commensal microbes in the gut influenced serum IgA levels, which protected mice against lethal sepsis when the intestinal barrier was damaged (110). Serum IgA was shown to target the same epitopes and used the same V-gene segments as plasma cells in the gut (111), suggesting that systemic and mucosal plasma cells originated from the same B cell clone. Diminished serum IgA titers were found in GF mice compared to specific pathogen free mice, supporting the essential role for microbiota diversity in IgA production (110, 112). Mice lacking pIgR and SIgA have epithelial barrier disruption, enhanced numbers of IgA-secreting plasma cells and systemic immune activation indicated by increased levels of serum IgG and IgA (113, 114). Higher levels of albumin were found in the feces, suggesting leakage of serum proteins across the epithelium (113). At the same time intestinal proteins and microorganisms can leak into the tissue and enter the bloodstream. After entering the portal circulation they will encounter Kupffer cells in the liver, which are specialized FcαRI^+^ macrophages that can eliminate IgA-coated bacteria from the bloodstream (Figure 2D) (34). Kupffer cells, macrophages, and monocytes produced increased levels of pro-inflammatory cytokines (e.g., TNF-α, IL-1β, and IL-6) after cross-linking of FcαRI in the presence of pathogen recognition receptor ligands (such as Pam3CSK4, LPS, flagellin) (reflecting IgA-opsonized pathogens) (115).

In contrast to the activating properties of complexed serum IgA, monomeric serum IgA was capable of downregulating cell responses and promoted powerful anti-inflammatory effects (Figure 2D). This is referred to as ITAMi signaling (see above) (19). Monomeric targeting of the FcαRI with serum IgA may protect against enhanced receptor activation, which is beneficial in inflammatory diseases characterized by the presence of IgG immune complexes or enhanced FcεRI signaling.

## IgA-associated Diseases

In babies circulating IgA levels are physiologically low because IgA cannot be transported across the placenta. Colostrum and breast milk are important sources of SIgA antibodies to provide local protection against infections. As soon as exposure to microbiota takes place, the SIgA immune system is rapidly maturing. Nonetheless, adult serum IgA levels are not reached before puberty as the systemic IgA compartment develops very slowly. The daily production rate of IgA exceeds that of all other combined antibody classes (66 mg/kg/day), suggesting that the role of IgA in immune defense must be considerable for the body to spend high energy levels (30). Abnormal IgA levels in serum and external secretions (either higher or lower) have been described in numerous pathologies. Very low or absent IgA is referred to as IgA deficiency (116). High levels of (aberrantly glycosylated) IgA are present in multiple diseases including IgA nephropathy, dermatitis herpetiformis, IgA vasculitis, and rheumatoid arthritis.

### IgA Deficiency

Selective IgA deficiency is the most common human immunodeficiency and is characterized by a serum IgA concentration of <7 mg/dL. Both serum IgA and mucosal IgA are very low or absent in selective IgA deficient patients (6, 117). Defects in B cell isotype switching, terminal differentiation of IgA^+^ plasma cells into secretory cells or long-term survival of IgA-secreting plasma cells can result in IgA deficiency (118, 119). Unbalanced cytokine production (including IL-4, IL-6, IL-7, IL-10, and IL-21) has been reported with a key role for TGF-β, as it induces isotype switching and differentiation of antigen-stimulated B cells into IgA-secreting plasma cells (120, 121). The initial defect may lie in the stem cell compartment as IgA deficiency can be transferred by bone marrow transplantation (122).

**Figure 3:**
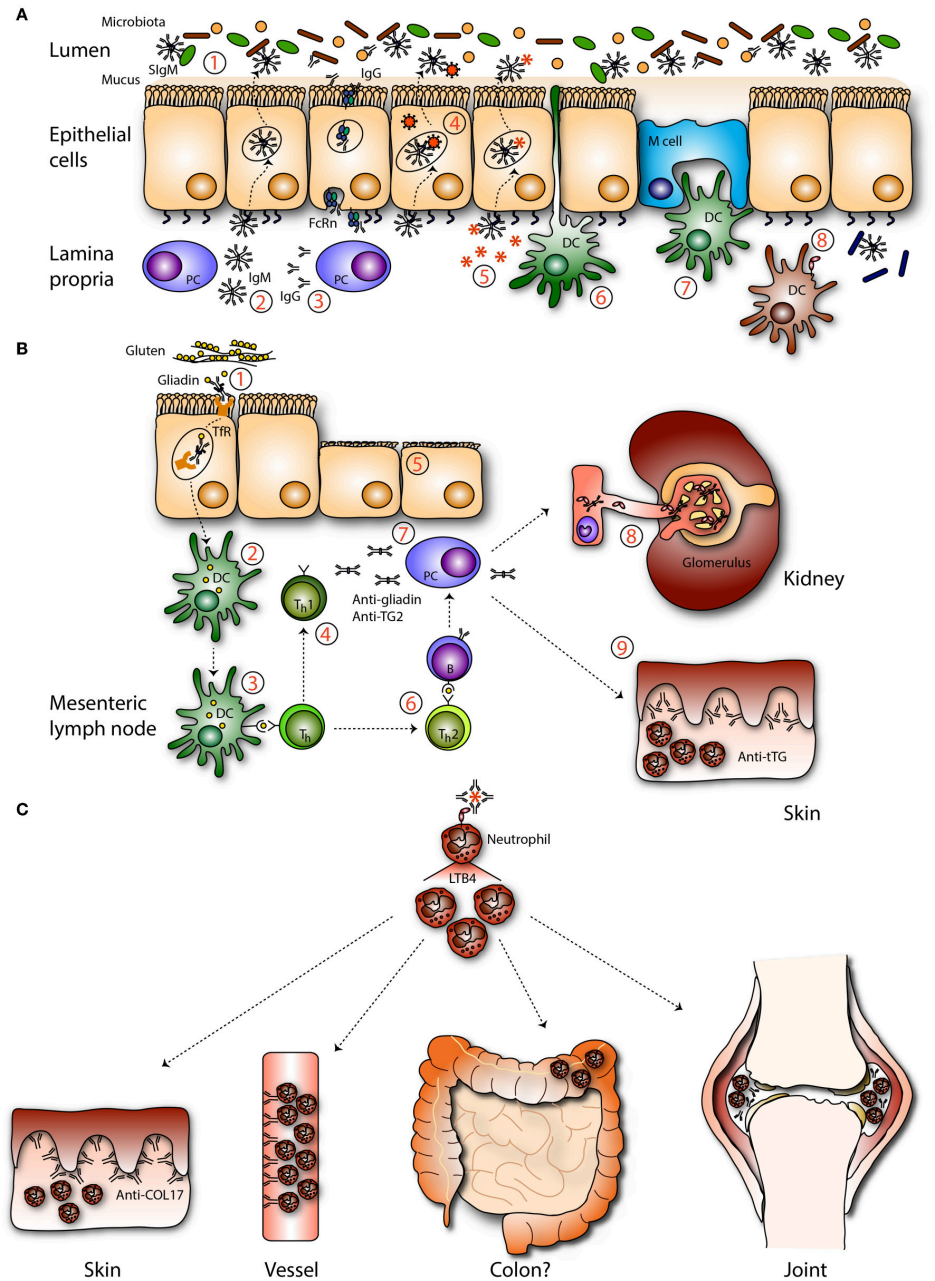
Mucosal IgA in pathogenesis. **(A)** IgA deficiency. In absence of IgA the intestinal microbiota is (1) less diverse and unbalanced. (2) IgM, and (3) IgG can be transported across the epithelium by associating with the pIgR or the neonatal Fc receptor (FcRn), respectively, to compensate IgA loss. Theoretically, IgM may be able to (4) neutralize viruses and (5) exclude infiltrated antigens. Sub epithelial DCs are not described to (6) sample IgM coated bacteria. (7) IgM-coated bacteria are not described to enter M cells. (8) Invaded IgM-coated pathogens are not recognized by FcαRI-expressing immune cells, resulting in less efficient pathogen elimination. **(B)** Celiac disease and gluten-associated diseases. (1) IgA-gliadin complexes bind to the transferrin receptor (TfR) and are retrotransported across the epithelium. (2) In the lamina propria deamidated gliadin is taken up by DCs and after processing (3) presented to T helper (Th) cells. (4) Th1 cell activation leads to the release of pro-inflammatory mediators (5) causing tissue damage. (6) Activated Th2 cells stimulate autoantibody production directed against gliadin and tissue transglutaminase by B cells. (7) Plasma cells produce autoantibodies which (8) can be detected in the circulation and form deposits with soluble FcαRI in the kidney resulting in damage (IgA nephropathy). (9) IgA anti-tissue transglutaminase forms complexes in the dermis, resulting in neutrophil activation and concomitant tissue damage (dermatitis herpetiformis). **(C)** IgA-FcαRI induced pathology. IgA immune complexes activate neutrophils by cross-linking FcαRI, resulting in release of LTB4 and enhanced neutrophil influx, which induces tissue damage as seen in the skin (LABD), vessels (IgA vasculitis), joints (RA), and potentially in colon (IBD).

A significant heterogeneity exists among IgA deficient patients. Many patients are asymptomatic and only few show minor clinical symptoms. This may in part be due to better hygiene, vaccination, and antibiotic use in modern Western society. Furthermore, it was shown that increased production of IgM and/or IgG can partially compensate for the lack of IgA (Figure 3A) (123, 124). IgA deficient patients can develop severe pathology when IgG and/or IgM does not compensate for IgA loss (117, 125). Conflicting reports on the influence of IgA deficiency on fecal microbiota in patients have been reported. One study showed a significantly less diverse fecal microbiota of IgA deficient patients compared to healthy controls, and specific taxa were lost in IgA deficient patients (124). Another study reported a mild loss in microbial diversity in IgA deficient patients (117). SIgM could partially rescue the microbiota composition in IgA deficient patients (117). However, SIgM targeted a broader range of microbes and showed less specificity for microbes compared to SIgA (124). Patients can have increased C reactive protein levels, which is indicative for enhanced inflammation, along with an increased risk of death 10–15 years after initial diagnosis (126, 127). In a recent study no risk association was found between IgA deficiency and hospital infections (127). IgA deficiency was also not more common among hospitalized individuals compared to healthy blood donors (123). However, patients have moderate enhanced susceptibility to gastrointestinal, urinary, and recurrent respiratory infections, allergies, celiac disease, and autoimmune diseases (6, 128, 129). Forty-five percent of IgA deficient patients have the 8.1 haplotype (HLA-A1, B8, DR3, DQ2 haplotype) compared to 16% in the general population (123, 130). The 8.1 haplotype is also commonly found in patients with autoimmune diseases (including rheumatoid arthritis and systemic lupus erythematosus), which are strongly associated with IgA deficiency (123). These findings suggest that patients share predisposing genes, which may explain the increased prevalence of selective IgA deficiency in certain autoimmune disorders.

Eighty-four percent of IgA deficient patients were positive for a wide variety of allergens in skin prick tests (131). The link between IgA deficiency and allergies may be explained by higher levels of circulating antigens due to increased permeability at mucosal surfaces. Alternatively FcαRI may not be able to induce ITAMi signaling in the absence of monomeric serum IgA, possibly resulting in overactivation of immune cells that can lead to development of allergies and autoimmune diseases (132).

Anti-IgA antibodies, usually of the IgG subclass, are commonly found in IgA deficient patients (40%), although their etiology remains unknown. Transfusing IgA-containing blood products to treat IgA deficiency is therefore complicated, as IgA-anti-IgA immune complexes can induce severe reactions, especially when anti-IgA antibodies of the IgE subclass are present (133, 134).

### Allergic Diseases

Allergic diseases are inflammatory conditions of which the majority are characterized by high specific IgE levels and activated mast cells (135). Alterations in microbiota diversity reduced immunological tolerance, potentially resulting in food allergies, allergic rhinitis, and asthma (136). It was suggested that the current hygienic life style in the western world altered normal microbiota colonization in infants thereby contributing to the enhanced prevalence of allergies (“hygiene hypothesis”) (137). A less diverse composition of the microbiota was associated with lower IgA levels, suggesting that IgA can be involved in the development of allergic diseases as well. Studies investigating the link between IgA levels and allergy and asthma have however been conflicting. In children, serum IgA levels correlated negatively with asthma severity (138). Moreover, it was shown that lower levels of intestinal bacteria were coated with IgA in infants with asthma (139). In adults, both house dust mite sensitization and airway hyper-responsiveness correlated negatively with serum IgA (140). Furthermore, asthma symptoms like shortness of breath and sputum production inversely correlated with, respectively mucosal SIgA and serum IgA levels (141). These results suggest a potential link between low IgA levels and the risk and severity of allergic asthma, which supports a protective role for IgA in allergy. However, data has also been reported showing enhanced specific IgA levels in patients with allergic rhinitis and atopic asthma (142, 143). Moreover, allergen specific IgA in combination with eosinophilia were common characteristics of asthma and allergic rhinitis (142), possibly because IgA can induce eosinophil survival, thereby contributing to disease severity (144). These results demonstrate a dual role of IgA in allergies, which is currently ill-understood.

### Rheumatoid Arthritis

Rheumatoid arthritis (RA) is a systemic and chronic autoimmune disorder characterized by infiltration of inflammatory immune cells in the joints, resulting in swelling and pain. Presence of autoantibodies including rheumatoid factor (RF) and anti-citrullinated protein antibodies are commonly present, which can be of the IgM, IgG or IgA isotype. High IgA anti-citrullinated protein antibodies and IgA RF titers correlate with worse disease prognosis and severity, and have been used as predictive value for disease progression (145–147). This suggests that IgA contributes to disease pathology. It was demonstrated that blocking the interaction between IgA RF and macrophage FcαRI resulted in reduced levels of TNF-α (148). Additionally, neutrophils stimulated with plasma of RA patients, containing IgA RF, induced NET release, which was inhibited by blocking FcαRI (149). Thus, IgA-immune complexes in patients can induce pro-inflammatory functions of neutrophils and macrophages, which are prominently present in inflamed joints (150), and as such contribute to inflammation in RA.

### IgA Nephropathy

IgA nephropathy (IgAN) is the most common form of glomerulonephritis and progresses to end-stage kidney failure in 50% of patients. IgA levels are increased in both serum and urine of IgAN patients. A link with RA was suggested, as patients can also have enhanced IgA RF in their serum. This condition is referred to as rheumatoid nephropathy (151). It was hypothesized that galactose-deficient O-glycans in IgA1, produced by plasma cells in the gut, can trigger the production of anti-glycan IgG/IgA antibodies, after which formed immune complexes deposit in the glomeruli where renal injury is initiated (152, 153). The enzymes responsible for production of O-linked glycans are glycosyltransferases, which expression are amongst others regulated by bacterial products, suggesting that the microbiome regulates the specific glycosylation pattern of IgA1 during the initial phase of IgAN (154, 155). In mice, galactose-deficient IgA1 was not cleared from the murine circulation and deposited in the kidney (15). FcαRI^+^ Kupffer cells were unable to clear circulating IgA of IgAN patients *in vivo* (156). In transgenic mice expressing the human FcαRI on monocytes and macrophages, soluble FcαRI-IgA complexes that were deposited in the mesangium, induced glomerular and interstitial macrophage infiltration, hematuria, mesangial matrix expansion, and mild proteinuria (157, 158). Moreover, in FcαRI transgenic and human IgA knock-in mice soluble FcαRI-IgA complexes induced kidney inflammation by interacting with TfR1 on mesangial cells, which induced the release of pro-inflammatory mediators. Additionally, the expression of transglutaminase 2 was induced, which subsequently enhanced the expression of TfR1, thereby inducing a pathogenic amplification loop (157). Of note, shedding of FcαRI from macrophages of transgenic mice may have aggravated disease, which will likely not occur in patients as human macrophages express lower FcαRI levels. Nonetheless, IgA-soluble FcαRI complexes have been found in patients with IgAN, and serum IgA immune complexes bound more avidly to TfR1 *in vitro* compared to those from healthy controls (158, 159). It was demonstrated that IgAN and Henoch-Schönlein purpura nephritis patients had reduced levels of soluble FcαRI and transglutaminase 2 in their urine (160), which makes it plausible that soluble FcαRI immune complexes deposit in the human kidney.

### IgA Vasculitis

IgA vasculitis, also known as Henoch-Schönlein purpura, is the most common form of vasculitis. The condition is characterized by IgA1 immune deposits and neutrophil infiltrates affecting the small vessels. As a result, red blood cells can leak into the skin leading to typical cutaneous hemorrhages, which leads to diagnosing the disease (161, 162). The pathology of IgA vasculitis remains unclear. However, it is suggested to represent a systemic equivalent of IgAN, as IgA vasculitis can be accompanied with nephropathy, resembling IgAN (163). Unlike IgAN, which is characterized by deposits of galactose-deficient IgA1, it is unknown which type of IgA accumulates in IgA vasculitis (152, 164).

Increased levels of soluble FcαRI-IgA complexes were found in sera of adult and pediatric vasculitis patients with or without co-existing nephritis, which was associated with decreased FcαRI expression on monocytes (165, 166). Furthermore, it was proposed that IgA1 anti-endothelial cell antibodies might play a role. Serum IgA from IgA vasculitis patients was shown to bind *in vitro* to human but not bovine glomerular endothelial cells (167). IgA anti-endothelial cell antibodies induced the production of IL-8 by endothelial cells, thereby contributing to an inflammatory environment and neutrophil recruitment (168, 169). In addition to enhanced levels of serum TNF-α, which promoted anti-endothelial cell antibody binding to endothelial cells, IL-8 production increased inflammation in IgA vasculitis patients (167, 169, 170). It was hypothesized that neutrophils become activated by IgA-FcαRI mediated cross-linking, resulting in inflammatory processes like reactive oxygen species production and NET formation. Moreover, IgA-activated neutrophils release LTB4, inducing neutrophil migration, thereby enhancing a positive feedback loop, which may result in pathogenesis observed in IgA vasculitis (162).

### Inflammatory Bowel Disease

Inflammatory bowel disease is characterized by chronic inflammation in the intestinal tract and is subdivided into ulcerative colitis and Crohn's disease (171). The barrier function in these patients is disrupted allowing bacteria to invade the subepithelial lamina propria (171). Downregulation of pIgR on intestinal epithelial cells and a disturbed microbiota were observed in IBD patients (172). It was proposed that intracellular signaling pathways and protein trafficking was altered due to damage caused by the inflammatory environment resulting in pIgR regulation defects (10). Mice lacking pIgR have enhanced numbers of IgA-secreting plasma cells, possibly as compensation. Increased production, combined with diminished transcytosis of dIgA into the lumen may potentially result in accumulation of dIgA in the lamina propria. Invading bacteria can become opsonized with dIgA, potentially resulting in activation of neutrophils by cross-linking of FcαRI, leading to tissue damage. Neutrophils that had taken up IgA were observed in the mucosa of ulcerative colitis patients (64). Cross-talk between Fc receptors and TLRs was induced by antibody-coated bacteria, resulting in release of pro-inflammatory mediators (e.g., LTB4) by DCs, which may also contribute to inflammation in IBD. LTB4 induces recruitment of neutrophils and monocytes. Additionally, simultaneous triggering of FcαRI and TLR4 on neutrophils resulted in enhanced release of pro-inflammatory TNF-α (173). Furthermore, due to diminished SIgA levels in the lumen, neutralization, and immune exclusion of microbes is decreased, which may worsen disease in patients (10). Patients have increased levels of specific IgA against microbiota in their serum (174). Highly IgA-coated bacteria obtained from IBD patients induced colitis in GF mice (175). The extent to which IgA responses against microbiota differ in homeostatic conditions vs. IBD, remain unclear (86).

Surprisingly, an increased amount of fecal bacteria of IBD patients was opsonized with IgA compared to those obtained from healthy control feces (85, 175, 176). It was suggested that leakage of serum IgA or dIgA due to a disrupted epithelial layer contributed to enhanced fecal bacteria coating (176, 177). The exact role of IgA and FcαRI in inflammatory bowel disease however remains to be elucidated.

### Celiac Disease and Dermatitis Herpetiformis

Celiac disease is a multifactorial autoimmune disease characterized by a damaged small intestinal mucosa and nutrient malabsorption following gluten ingestion. Individuals carrying the DQ2 and DQ8 HLA haplotypes have an increased risk to develop celiac disease (178). Gliadin, a glycoprotein of gluten, can complex with specific IgA, after which it can be retrotransported across the epithelium via the TfR (Figure 3B). Celiac disease patients have increased expression of TfR on their epithelial cells, allowing increased retrotransport (12). Intracellular degradation of these peptides is disturbed in patients and after entrance in the lamina propria gliadin peptides are deamidated by tissue transglutaminase after which they are presented to HLA-DQ2 or HLA-DQ8 expressing CD4^+^ T cells. This results in the release of pro-inflammatory mediators, concomitant tissue damage and production of autoantibodies (179). IgA anti-tissue transglutaminase antibodies play a key role in disease pathogenesis (180). Presence of these antibodies serve to diagnose the disease and are furthermore linked to other IgA-related diseases like dermatitis herpetiformis and IgA nephropathy (Figure 3B). In dermatitis herpetiformis aberrant IgA antibodies are directed against structural proteins maintaining cell-cell adhesion in the epidermis leading to skin tissue damage (181). It is hypothesized that celiac disease patients can develop dermatitis herpetiformis as high avidity IgA anti-tissue transglutaminase antibodies form immune complexes and deposit in the dermis of these patients, although it is ill understood why some patients develop dermatitis herpetiformis, and others do not. Furthermore, small bowel inflammation in celiac disease is mostly the result of mononuclear cell infiltration, whereas neutrophils accumulate in the skin of dermatitis herpetiformis patients (181). Patient neutrophils have increased ability to bind IgA without FcαRI expression alteration, supporting that FcαRI is primed (e.g., by cytokines or differential glycosylation) (182).

Gluten exposure can also lead to glomerular IgA deposition and IgAN (183). Transgenic human IgA and FcαRI knock-in mice developed severe pathology when gluten were present in their diet, including intestinal injury, increased IgA1–soluble FcαRI complexes, mesangial IgA1 deposition, and elevated serum IgA1 anti-gliadin antibodies. IgAN patients also have increased IgA1 anti-gliadin antibodies in their serum, suggesting that entrance of gluten can potentially result in loss of oral tolerance thereby contributing to IgAN (184). Consumption of a gluten-free diet can resolve manifestations of celiac disease, dermatitis herpetiformis, and IgAN.

### Linear IgA Bullous Disease

Linear IgA Bullous Disease (LABD) patients suffer from extensive skin damage and blister formation caused by the presence of IgA autoantibodies against collagen XVII, and concomitant neutrophil accumulation (Figure 3C). Collagen XVII plays a critical role in maintaining adhesion between dermis and epidermis in the skin (185). Neutrophil activation is likely the direct result of FcαRI triggering by IgA autoantibodies (186). Tissue damage was induced in the presence of neutrophils and serum of LABD patients (containing IgA anti-collagen XVII autoantibodies) in an *ex vivo* skin model, as presence of activated neutrophils led to separation of the dermis from the epidermis (reflecting blister formation) (186). Eosinophil influx has been observed in the skin of LABD patients as well, suggesting that these cells might also contribute to disease pathology through FcαRI mediated respiratory burst activity (185).

## IgA and FcαRI as therapy

**Figure 4:**
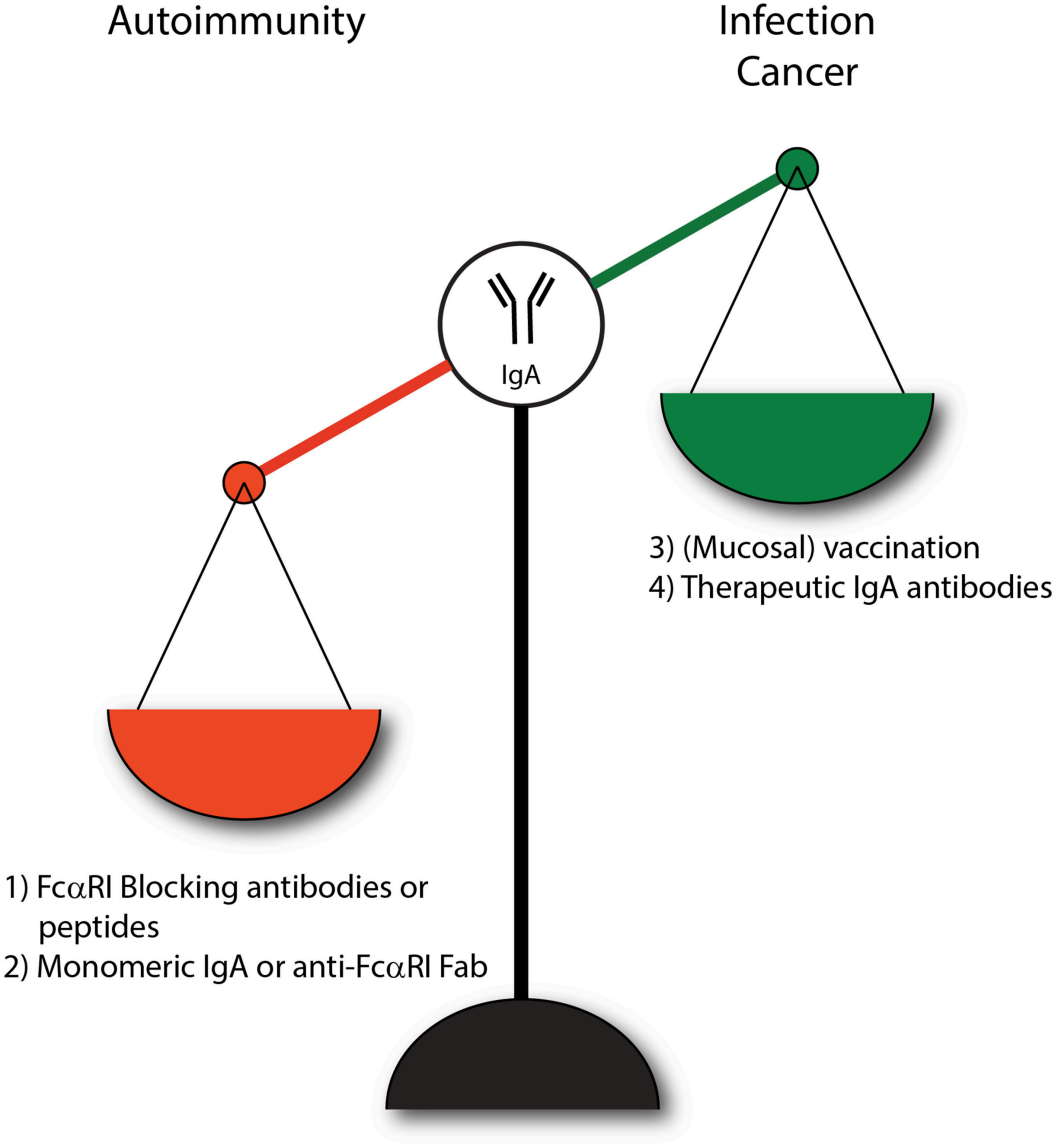
IgA and FcαRI as therapeutic targets. (1) Enhanced IgA-FcαRI activation in IgA-associated autoimmune diseases is unwanted. Blocking IgA-FcαRI interactions by monoclonal antibodies or peptides may reduce tissue damage in these diseases. (2) Treatment with monomeric IgA or anti-FcαRI Fabs may induce ITAMi signaling thereby inhibiting IgG-induced phagocytosis and IgE-mediated allergic diseases. (3) To combat infections, administration of IgA (passive vaccination) or induction of IgA levels via active vaccination may result in enhanced protective immunity. (4) IgA monoclonal antibody therapy may result in efficient killing of tumor cells by activating FcαRI-expressing immune cells.

IgA plays an important role in dampening mucosal infections, but can also have detrimental effects in inflammatory or autoimmune diseases. Lack of IgA may increase susceptibility to infection, whereas overabundant IgA complexes or autoantibodies can be harmful as enhanced activation of the FcαRI may lead to various pathologies. Several therapeutic strategies have been proposed that influence the IgA-FcαRI axis (Figure 4). For instance, in order to benefit from increased IgA levels during infections it was proposed to increase pathogen-specific IgA levels through passive or active vaccination thereby resulting in efficient clearance of pathogens. Furthermore, IgA might be used as a therapeutic antibody to mediate efficient tumor cell killing. Additionally, two different strategies of targeting FcαRI have been proposed. First, the induction of ITAMi signaling via FcαRI may dampen inflammation that is caused by other pro-inflammatory receptors (e.g., FcεRI). Second, blocking FcαRI may reduce activation of immune cells in IgA-mediated inflammation.

### IgA in Vaccination Strategies

Administration of specific IgA (passive immunization) or enhancing IgA production through active immunization might be effective strategies to combat viral and bacterial infections (104). Intranasal passive administration of either specific monomeric or polymeric IgA against a mycobacterium tuberculosis antigen led to short, but effective protection against infection in mice (187). The short protection duration was probably due to degradation of IgA by bacterial proteases present in the respiratory tract fluid. Furthermore, mice expressing human FcαRI on blood neutrophils and monocytes had a lower mycobacterium tuberculosis infection rate compared to control mice after inoculation of human IgA mAb, supporting an additive beneficial role for FcαRI in clearing the infection (188). Wild type (WT) mice which orally received *Salmonella typhimurium* that had been complexed with human plasma-derived IgA and IgM had reduced intestinal infection rates compared to mice exposed to *Salmonella typhimurium* only. Enhanced bacterial clearance was observed when SC was coupled to plasma-derived IgA and IgM (189). Polymeric IgA against influenza virus reached the nasal mucosa after intravenous administration and protected WT mice against infection, and was 10 times more effective than IgG in reducing viral shedding (190, 191). Passive immunization with recombinant dIgA showed better prevention against intra-rectal simian HIV transmission in rhesus macaques. Dimeric IgA could either directly neutralize simian HIV or the virus was trapped by large immune complexes preventing entrance in the epithelial barrier (103). It was demonstrated that IgA isolated from serum of HIV survivors and vaccinated HIV patients neutralized HIV through occupying the CD4 binding site of the virus (192, 193). By contrast, in another study, HIV specific IgA levels in plasma interfered with IgG effector functions *in vitro*, thereby increasing HIV infection risk (194). Recently, it was reported that oral and nasal administrated HIV antigens bound to SIgA were retro transported through M cells and reached sub-epithelial DCs, thereby inducing both mucosal and systemic humoral and cellular immune responses in CX3CR1/GFP transgenic mice (195). Vaccination with SIgA-HIV antigen protected these mice from infection after challenge with a recombinant vaccinia virus expressing the HIV antigen (195, 196). This demonstrates the potential of SIgA to serve as a vaccine carrier for HIV via mucosal administration.

Vaccination with live attenuated polio induced intestinal IgA production and induced long-lived memory immune responses in elderly who still had detectable serum and salivary IgA levels (197–199), which suggested that priming with live virus is needed to obtain IgA responses after booster vaccination with inactivated polio virus. Furthermore, WT mice that were immunized and re-challenged with reovirus had enhanced serum and intestinal IgA levels and were protected from infection (108, 200). IgA^−/−^ mice were unable to clear reovirus infection (108). Mice immunized with hemagglutinin (surface protein on influenza virus) produced anti-influenza specific SIgA, which protected mice from infection after intranasal administration (201). Mice deficient of IgA, J-chain or pIgR showed compromised immune protection (106–108), supporting that IgA enhances protective immunity against viral and bacterial infections.

### FcαRI as Therapeutic Target in Cancer Antibody Therapy

Antibodies targeting specific tumor-associated antigens like epidermal growth factor receptor (EGFR) and human epidermal growth factor two (HER2) are increasingly used to treat solid tumors (respectively colorectal cancer and breast cancer) (202). These therapeutic antibodies are often of the IgG isotype and have long half-life, can activate complement, and recruit natural killer cells as well as macrophages as cytotoxic effector cells (202, 203). Alternatively, IgA or FcαRI bispecific antibodies (BsAb) may represent promising novel drugs to treat cancer by enhancing activation of FcαRI-expressing immune cells (204). For instance, BsAb targeting both tumor antigens and FcαRI efficiently recruited neutrophils *in vitro*, which was not observed after targeting Fcγ receptors (205–207). Moreover, (immature) neutrophils were able to kill tumor cells more efficiently due to FcαRI-induced antibody dependent cellular cytotoxicity (62, 203, 208). Similarly, IgA anti-tumor mAbs mediated tumor killing more efficient compared to IgG mAbs. *In vitro* studies demonstrated the superior ability of FcαRI to induce neutrophil-mediated tumor cell killing for multiple tumor antigens, including EGFR, HER2, EpCAM, HLA-II, CD20, CD30, and carcinoembryonic antigen (203, 205, 209).

Unfortunately, *in vivo* tumor targeting using IgA anti-tumor mAbs has been difficult as mice do not express a homolog of the human FcαRI, and human IgA has a short half-life in mice (158). The generation and utilization of FcαRI transgenic mice (71, 158) can contribute to study IgA as therapeutic antibody *in vivo*. Treatment with IgA anti-HER2 or IgA anti-EGFR anti-tumor mAbs resulted in significantly enhanced anti-tumor cytotoxicity in FcαRI transgenic mice compared to WT littermates, which was mediated by FcαRI-expressing macrophages (210, 211). Furthermore, IgA anti-CD20 mAbs inhibited B cell lymphoma cell proliferation *in vitro* and recruited FcαRI-expressing immune cells *in vivo* in FcαRI transgenic mice (212, 213).

Thus, IgA antibodies have been shown to recruit neutrophils and macrophages as effector cells, but the short half-life of IgA in serum is a major drawback for use of IgA as therapeutic antibody. Compared to IgG, the glycosylation sites of therapeutic IgA mAbs that are produced in non-human systems (like rodent cells) have higher immunogenic potential (high glycosylation) and therapeutic IgA will be likely cleared efficiently from the human (or murine) circulation. Increasing the sialylation of glycans on IgA mAbs enhanced their serum half-life due to decreased clearance by the ASGPR in the liver (210). Inducing sialylation of IgA mAbs was done by using fully human systems thereby augmenting their *in vivo* therapeutic potential (211, 214–216). Increased sialylation of the N-glycans in IgA anti-HER2 mAbs resulted in significantly reduced tumor growth in FcαRI-transgenic SCID mice bearing BT-474 tumors (214). Addition of an albumin-binding domain to IgA1 (IgA1-albumin) enhanced interaction of the antibody with the FcRn, which extended the half-life of IgA1 *in vivo*. Although *ex vivo* studies showed that IgA1-albumin induced lower maximal tumor cell lysis, enhanced tumor cell killing was observed *in vivo* (211). Another modified IgA molecule against EGFR lacking glycosylation and free cysteine's, and with a stabilized heavy and light chain linkage, showed increased efficacy by interacting with FcαRI-expressing myeloid cells *in vivo*. Some therapeutic activity was observed in non-FcαRI transgenic mice demonstrating contribution of Fab-mediated effector functions as well (216). Although IgA antibodies are not used in clinical studies yet, engineering IgA with increased half-life represents a promising strategy for targeting tumors in patients.

### Induction of ITAMi Signaling

Enhancing ITAMi signaling by monomeric targeting of the FcαRI may be a promising strategy to inhibit IgG-induced phagocytosis and IgE-mediated allergic diseases (20). Monovalent targeting of FcαRI with the anti-FcαRI mAb A77 inhibited degranulation of RBL-2H3 transfected cells and reduced airway inflammation in FcαRI transgenic mice after crosslinking FcεRI with IgE immune complexes in an allergic asthma model (20, 217). Furthermore, naturally occurring serum IgA dampened immune responses by inducing ITAMi signaling via FcαRI (19). FcαRI transgenic mice (expression on monocytes/macrophages), which developed RA after injection with IgG anti-collagen had reduced manifestations or even complete resolution of arthritis after treatment with monomeric IgA. IgG-induced ITAM signaling was blocked efficiently, implicating a potential role of monomeric IgA in treatment of autoimmune diseases with IgG autoantibodies (218). Moreover, prior targeting of FcαRI by Fab A77 suppressed inflammation in transgenic mice with FcαRI-expressing monocytes and macrophages that suffered from IgG immune complex glomerulonephritis and obstructive nephropathy (219). Renal inflammation induced by pristane was characterized by enhanced serum IgG levels, pro-inflammatory cytokines and immune infiltration in FcαRI transgenic mice with receptor expression on monocytes and macrophages. Blocking FcαRI with Fab MIP8a inhibited cytokine production, leukocyte recruitment and inflammation (77). Furthermore, renal inflammation induced by CpG (TLR9 agonist) in these FcαRI transgenic mice was downregulated by monomeric occupancy of FcαRI (220). Thus, monomeric targeting of FcαRI is suggested to induce anti-inflammatory properties, which could be useful in treatment of inflammatory diseases with involvement of myeloid cells.

### FcαRI Blocking

Blocking FcαRI might be effective in IgA-mediated inflammation. *In vitro* it was shown that neutrophils stimulated with IgA immune complexes obtained from RA patients released neutrophil extracellular traps. FcαRI blocking on neutrophils with the anti-FcαRI mAb MIP8a reduced NET formation and might alleviate neutrophil induced tissue damage in RA patients (149). IgA autoantibodies in serum of LABD patients induced neutrophil-mediated tissue damage in an *ex vivo* human skin model. FcαRI blocking with MIP8a prevented IgA induced tissue damage (186). Similarly, peptides targeting the interaction sites of IgA and FcαRI showed effective blocking of IgA binding to FcαRI, and reduced IgA-induced neutrophil migration *in vitro*. These peptides were able to penetrate into human skin, supporting that they might function as novel therapy in skin autoimmune diseases (221). As such, blocking FcαRI might serve as therapeutic strategy for IgA-associated inflammatory diseases.

## Conclusion

IgA is important in maintaining balance of mucosal immunity. SIgA regulates immune exclusion by neutralizing pathogens and more recently the role of SIgA in diversifying the intestinal microbiota has become clear. The mechanisms involved in IgA-mediated regulation of microbiota diversity and reciprocal regulation of IgA levels by microbes are incompletely understood. Moreover, it remains unclear why certain SIgA coated antigens associate with the epithelial layer while others are eliminated via immune exclusion. In addition to the traditional role of IgA as non-inflammatory regulator of homeostasis, several pro-inflammatory functions have been described, which need to be clarified in more detail.

After binding to FcαRI, IgA plays important roles in pathogen elimination. It can also have detrimental effects on human health when aberrant IgA is present. Perpetual IgA-FcαRI interaction results in enhanced activation of immune cells with concomitant tissue damage as seen in autoimmune diseases like LABD (64). It is yet undefined why neutrophil influx is observed in some IgA-FcαRI mediated diseases, including LABD and dermatitis herpetiformis, but not others. For instance, Crohn's disease is generally less characterized by neutrophil influx in the intestinal mucosa, while in patients with ulcerative colitis massive neutrophil infiltration is present (171). Similarly, and maybe even more confusing, is the fact that celiac disease patients develop IgA anti-tissue transglutaminase antibodies resulting in infiltration of mainly mononuclear cells in the intestinal tract, while in dermatitis herpetiformis (skin manifestation of celiac disease) binding of IgA autoantibodies to epidermal transglutaminase results in neutrophil recruitment (181).

Targeting the IgA-FcαRI axis with blocking monoclonal antibodies or peptides may alleviate inflammation and concomitant tissue damage. Moreover, inhibitory ITAMi signaling induced by monomeric targeting of FcαRI has been suggested to represent a promising strategy in allergies or IgG immune complex-mediated diseases. By contrast, in infectious diseases and cancer enhancing pro-inflammatory effects of IgA-FcαRI interaction might be very beneficial.

Lack of an FcαRI equivalent in mice has hampered our understanding of the functions and therapeutic applications of IgA and FcαRI. Future research will be facilitated by the use of several human FcαRI transgenic and human IgA knock-in models (71, 158, 222). This will help to increase our knowledge on the complex roles of IgA and FcαRI in (patho)physiology as well as the therapeutic possibilities for targeting these multifaceted molecules.

## Author Contributions

AB wrote the paper and designed the figures. MvE designed and illustrated the figures, supervised, and revised the paper.

### Conflict of Interest Statement

The authors declare that the research was conducted in the absence of any commercial or financial relationships that could be construed as a potential conflict of interest.

## Abbreviations

ASGPR: Asialoglycoprotein receptor
BsAb: Bispecific antibodies
DC-SIGN: Dendritic Cell-Specific Intercellular adhesion molecule-3-Grabbing Non-integrin
DCs: Dendritic cells
dIgA: Dimeric IgA
FcαRI: Fc alpha receptor
FcRL4: Fc receptor-like 4
fMLP: N-formylmethionyl-leucyl-phenylalanine
GF: Germ free
GM-CSF: Granulocyte-macrophage colony–stimulating factor
IBD: Inflammatory bowel disease
IFN-γ: Interferon-gamma
IgA: Immunoglobulin A
IgAN: IgA nephropathy
IL: Interleukin
ITAM: Immunoreceptor tyrosine-based activation motif
ITAMi: Inhibitory immunoreceptor tyrosine-based activation motif
kDa: Kilodalton
LABD: Linear IgA Bullous disease
LPS: Lipopolysaccharide
LTB4: Leukotriene B4
M cells: Microfold cells
pIgR: Polymeric immunoglobulin receptor
RF: Rheumatoid factor
SC: Secretory component
SIgA: Secretory IgA
SIgM: Secretory IgM
Tfr: Transferrin receptor
TGF-β: Transforming growth factor-beta
TLR: Toll like receptor
TNF-α: Tumor necrosis factor-alpha.
